# General practitioners’ perceptions on home medicines reviews: a qualitative analysis

**DOI:** 10.1186/s12875-015-0227-8

**Published:** 2015-02-07

**Authors:** Amrith Kaur Dhillon, Hendrika Laetitia Hattingh, Andrew Stafford, Kreshnik Hoti

**Affiliations:** School of Pharmacy, Curtin University, Bentley, Perth, 6845 Australia

**Keywords:** Pharmacists, Home Medicines Reviews, General practitioners

## Abstract

**Background:**

Home Medicines Review (HMR) is an Australian initiative introduced in 2001 to improve quality use of medicines. Medication management services such as HMRs have the potential to reduce medication related problems. In 2011, changes to the HMR program were introduced to allow for referrals directly to accredited pharmacists in addition to the community pharmacy referral model. These changes were introduced to improve efficiency of the process. This study explored the perceptions of Western Australian general practitioners (GPs) on benefits and barriers of the HMR service and process, including their insights into the direct referral model.

**Methods:**

Purposive sampling of GPs who had experience ensured that participants had a working knowledge of the HMR service. Semi structured interviews with 24 GPs from 14 metropolitan Western Australian medical centres between March and May 2013. Transcribing and thematic analysis of data were performed.

**Results:**

Most GPs had positive attitudes towards the HMR service. Main perceived benefits of the service were poly-pharmacy reduction and education for both the GP and patient. Strategies identified to improve the service were introduction of a standard HMR report template for pharmacists and better use of technology. Whilst reliability and GPs’ familiarity were the main perceived benefits of the direct referral model, a number of GPs agreed that patient unfamiliarity with the HMR pharmacist was a barrier.

**Conclusions:**

Despite recognition of the value of the HMR service participating GPs were of the opinion that there are aspects of the HMR service that could be improved. As one of the success factors of HMRs is relying on GPs to utilise this service, this study provides valuable insight into issues that need to be addressed to improve HMR uptake.

## Background

There is an increasingly high prevalence of medication-related problems (MRP) in the Australian primary health care setting [1-5]. The government has therefore implemented a number of interventions to improve medication management services. Home Medicines Reviews (HMR) has been in place since 2001, introduced to improve quality use of medicines [6]. It is a collaborative medication review service that involves a referral from a general practitioner (GP) to a community pharmacy or an accredited pharmacist. The aim is to facilitate community-based patients’ medication therapy and minimise the prevalence of MRPs [7]. GPs plays a vital role in determining whether patients are likely to benefit from this service as HMRs require a referral from a GP. Previous literature indicated that some GPs believe HMRs potentially improve medication safety, awareness and management [8,9]. However, others have expressed concerns regarding the complexity of the HMR process, time constraints and inconsistency in the reporting format and quality [6,10,11]. Considering the variance in GP opinions towards HMRs, an understanding of their attitudes and beliefs is integral in addressing potential barriers in order to improve HMR uptake.

Prior to 2011, the only available option to refer a patient for a HMR was to the patient’s nominated pharmacy. This changed in 2011 whereby a second referral model directly to accredited pharmacists was introduced to improve efficiency of the process [12]. No research has been conducted to determine whether this “direct referral” model has improved HMR uptake.

Our aim was to explore Australian GPs' perceptions of the benefits and barriers of the initial HMR process and service as well as their perceptions of the new model. It was anticipated that a better understanding of GPs' attitudes towards the HMR service would inform the development of strategies to improve the HMR process. The objectives of this study were to obtain GPs’ perspectives and views on:

1. The role and value of HMRs,
2. The most beneficial aspects of the HMR service’
3. Strategies to improve the HMR process’ and
4. The direct HMR referral model to accredited pharmacists.

## Methods

Low-risk ethical approval was granted by the Human Research Ethics Committee of Curtin University (Approval PH-O1-13). This study involved semi-structured interviews with GPs practising in Perth, Western Australia (WA) who had utilised the HMR service. Purposive sampling of GPs who had experience ensured that participants had a working knowledge of the service which added value to the data. There were no pre-existing relationships between the researchers and the participants. This study was carried out by a final year pharmacy student (AD) under supervision of three pharmacy practice academics who were also experienced accredited pharmacists (LH, AS, KH).

Qualitative methodology was selected to allow for in-depth exploration of participants’ views [13]. A semi-structured interview guide was developed that addressed the study objectives consisting of the following nine questions:

1. How involved have you been in HMR program?
2. What do you feel the main aim of the HMR program is?
3. As a GP, describe the process you utilise in identifying whether a patient is likely to benefit from a HMR referral?
4. What aspects do you think are working well for this service?
5. What do you see as barriers that are limiting the uptake of this program?
6. What are your suggestions to improve the HMR process in specific to promote uptake, effectiveness and usefulness?
7. What sort of factors do you feel may influence the extent of service provided by GPs?
8. Explain your thoughts on the recent change that GPs are able to directly refer a HMR request to an accredited pharmacist rather than to a community pharmacy first?
9. Are there any more comments you would like to make regarding your experience with the HMR service that you would like to share?

Prompts allowed for re-wording, clarification and exploring of topics [14]. Open-ended questions encouraged GPs to discuss their perceptions of the role of HMRs, benefits, barriers limiting uptake and strategies for improvement. The final question provided an insight on their views of the new model. The interview guide was prepared by the primary researcher (AD) with guidance from the co-authors. The interview guide was tested on an academic practitioner for validation purposes. Interviews were conducted individually for practical reasons and GP time constraints.

GPs were recruited over a wide geographical area within Perth. Participant recruitment was initially through promotion of the project to medical centres and local practitioner networks. A total of 24 participants were set as the minimum target for reaching saturation [15]. The majority of participants were recruited by contacting 44 practice managers of medical centres. Additional GPs were approached through a snowball sampling technique [16] which involved recruitment through accredited pharmacists and participating GPs. Interviews were conducted at each GP’s practice. All participants were given an information sheet about the project and signed a consent form prior to the interview, also providing approval for audio recording. Participants received a small gift voucher.

Transcribing and thematic analysis of data were done manually by the primary researcher (AD) using Word 2010 (Microsoft Corporation, Redmond, United States). This involved an in-depth process of colour-coding themes and sub-themes. Data collection ceased when saturation was achieved. The themes identified emerged from the in-depth analysis of the interview data. All transcribed interviews and data analysis were reviewed by the research team members independently to confirm consistency.

## Results

A total of 24 GPs (19 male, 5 female) from 14 metropolitan medical centres in Perth were interviewed between March and May 2013. Interviews ranged from 12- 35 minutes (mean 21 minutes +/- 5.7). Seventeen of the GPs were aware of the direct referral model and 12 had utilised this option. Thematic analysis of the interview transcripts highlighted several areas of interest. There were four main themes that emerged namely:

- Patient and GP benefits,
- Barriers limiting uptake,
- Strategies for improving the HMR process and
- Benefits of the direct referral model to accredited pharmacists.

### Patient and GP benefits

The majority of participants expressed support towards the HMR service. Table 1 is a summary of selected comments by participants expressing patient and GP related benefits.

**Table 1:** **GP comments about HMR benefits**

**Benefit**	**Comment**
**Patient related benefits**	
Education on the need of medication	*“So again advantages for patients, I think it’s fairly straight forward that the patients become aware of every medication they are taking.”* (GP3)
Poly-pharmacy reduction	*“Ever since I started using HMRs, I realised instead of having multi-drug therapy, we can cut it down to four. From say eight, we can cut it down to six, four and that’s fantastic because patient compliance became much better and our drug interaction became less and patients wellness became much better.”* (GP6)
Preventing interactions between potentially harmful combination of medicines	*“Ensuring no dangerous drug interactions is potentially being missed.”* (GP10)
Education on medication administration	*“Well better education of the patient in terms of how they should be utilising their medications.”* (GP20)
Preventing confusion with generic medication	*“What we want to do is to confirm that at home that the patient is taking the regimen we believe they are so that there is no accidental confusion with the variety of generics medications which are doubling up on doses.”* (GP8)
Reassurance mechanism	*“I think it enhances the patient’s engagement with the health system and they feel like somebody cares which is good and also I think it enhances the patient’s feeling of responsibility of self-care.”* (GP11)
Education on side effects of medicines	*“Just makes the patient more knowledgeable and to look out for any side effects.”* (GP13)
Optimising cost	*“It also optimises the cost for the patient.”* (GP19)
**GP related benefits**	
Reconfirms current complementary, OTC and other medication	*“It brings to the fore other issues like OTC medication which is a reality of what we live in. People can get all sorts of medication, some not very good while some are beneficial like complementary medication, naturopathic medications and so when you conduct a Home Medicines Review; it brings those things to the fore.”* (GP19)
Preventing interactions between prescribed and complementary medicines	*”Trying to make sure patients are not taking herbal or other complementary medicines that’s going to interact with what they are taking.”* (GP5)
Reassurance mechanism	*“I can also feel a bit more comfortable that with the support of a pharmacist reviewing what’s happened at home that what I’ve heard the pharmacist has identified; we are all on the same page.”* (GP1)
Education on side effects associated with current medication and suggests relevant monitoring tests for patients	*“The feedback I get is a learning curve for me because there’s a lot of new knowledge gained from the accredited pharmacist looking at side effects or monitoring.”* (GP3)
Improving dosage knowledge in patients with chronic renal disease	*“I have had feedback particularly with medicines associated with people with chronic renal disease as to doses and that’s some good stuff.”* (GP8)

### Patient related benefits

The majority of participants agreed that HMRs facilitate compliance through educating patients about the correct use of medicines. Participants also indicated that this service could serve as reassurance for patients, thereby increasing self-confidence and independence. Additionally, some participants stated that HMRs could assist patients in the monitoring for side effects associated with particular medicines and medication administration. Participants also indicated that HMRs were specifically useful in patients with cognitive impairment and immigrants with a poor command of English. A reduction of costs for patients was another perceived benefit.

### GP related benefits

A number of participants stressed that HMRs improved their pharmaceutical knowledge, commenting that after receiving a HMR report they were more informed of patients’ complementary, over the counter (OTC) and prescription medication. Participants highlighted that HMRs provided insight into potentially harmful combinations of medicines that patients take without GPs’ knowledge. This allowed for concerns regarding potentially harmful combinations to be addressed. The decrease in poly-pharmacy through a reduction in unnecessary medication was addressed by 19 participants. Additionally, issues pertaining to duplication of therapy due to generic substitution were also addressed.

Participants mentioned that upon completion of a HMR they were able to make better judgments as to whether a patient required any additional medication monitoring. They were also able to address issues associated with side effects of medication. Two participants stated that HMRs improved their knowledge of medication dosing in patients with chronic renal disease. HMRs were also found to reassure GPs that patients were being managed appropriately. Based on previous experience, two participants reported higher utilisation of HMRs in retirement villages and country (rural) settings, indicating specific benefits in these settings.

### Barriers limiting uptake

Table 2 is a summary of statements by participants identifying the barriers reported.

**Table 2:** **GP comments about HMR barriers**

**Barrier**	**Comment**
**Patient related barriers**	
Discomfort due to intrusion of privacy in domestic setting	*“Sometimes patients may not want to have somebody come to their house and that might be for a whole range of reasons and it could possibly indicate that there is an issue acting on the domestic front that they feel uncomfortable and this is something that needs to be concerned about.”* (GP8)
Lack of awareness on HMR effectiveness	*“They don’t fully understand why they need a HMR or how it will benefit them; they are then less likely to go for it.”* (GP10)
**GP related barriers**	
Long drawn-out process and tedious paperwork	*“The other scenario is the mindset that is it an administrative nightmare. You have to do the paperwork and comply with Medicare requirements. There’s a lot of time involved pre and post, you have to read the thing, a lot of doctors just say “oh forget it”. I might as well do consults and leave it there.”* (GP1)
GP awareness	*“We were not very well exposed to HMRs. Most of our patients are well controlled but we never looked at it as an option as to why the patient needs a HMR or if a patient’s illness was not controlled, we always think of referring to a specialist or we’ll think of other options.”* (GP6)
Time constraints	*“For me, it’s probably just being busy to be honest with you.”* (GP21)
	*“I think the program has got a lot of merit but I’m not doing them because I’m time poor. Although I think it is a great idea and it is beneficial for patients, I just don’t seem to be able to incorporate them into my busy life.”* (GP 15)

### Patient related barriers

Participants reported that patient discomfort associated with the HMR being conducted at their residence resulted in resistance to the service. A lack of patient awareness regarding the effectiveness of HMRs was also reported.

### GP related barriers

A few participants expressed lack of interest in using the HMR service and one stated that HMRs were not beneficial. The long convoluted HMR process, tedious paperwork, time constraints, and low awareness of the HMR service were identified as the four main barriers limiting HMR uptake. One participant highlighted the lack of information available on patient eligibility.

### Strategies to improve the HMR process

Participants suggested that the process of HMR could be improved if accredited pharmacists used a standard HMR report template and focus on practical recommendations. The incorporation of an electronic reporting program was described by participants as another strategy to improve HMR uptake. A minority believed that assigning specific personnel from the practice to be the focal point of the program could contribute to the efficiency of the process. Supporting statements are reflected in Table 3.

**Table 3:** **GP focused strategies on approaches to improve the HMR process**

**Suggestion**	**Comment**
HMR template for pharmacists	*“I think they should have a standard format, just fill in the blanks. These are the drugs, these are the interactions right, suggestions one, two, three, four.”* (GP6)
	*I’ve noticed … most of the pharmacists have a very easy to read format. There are one or two pharmacists write their response in the form of an essay that’s a bit difficult to absorb … so obviously if they come out with a universal way of doing this, that will be ideal*. (GP3)
	*Generalised template with the same information but less writing out of information. I think that would suit doctors better because a lot of the cut and paste we already know*. (GP10)
Electronic software	*“The more that it becomes an online process, it would certainly help the situation cause that just speeds things up and if they send it back online, I think that process would definitely help.”* (GP7)
Assigning personnel from practice to be the focal point of the program	*“This is why I think it’s important to have somebody not a GP in the practice to run the HMR process because GPs are too busy.”* (GP11)

### Benefits of the direct referral model to accredited pharmacists

Benefits were mostly associated with reliability and GP familiarity with the pharmacist. The following statement illustrates this:

“*I think if you establish a rapport with one or a number of pharmacists that you have confidence in their work quality and you’re happy to use it because you know what you are getting back will be worthwhile.”* (GP 8)

However, some GPs had concerns about the fact that patients may not know the accredited pharmacist specifically if the accredited pharmacist was not their community pharmacist. These GPs were more inclined towards patient preference in the choice of the pharmacist conducting the HMR.

## Discussion

This study provided insights into GPs’ opinions towards HMRs. While a range of views were expressed, most of the participants had a positive attitude towards the program. The majority of the GPs were of the opinion that HMRs were beneficial to patients’ medication understanding and management. The GPs highlighted the medication management advantages from their perspectives as well as educational benefits for them as well as their patients. Barriers limiting HMR uptake mainly involved issues with HMR processes and GPs’ time constraints. Potential strategies for improving the HMR process included a standard HMR report template and an electronic reporting system.

This is the first published study to provide insight into GPs’ perceptions of the direct referral model. GPs noted that reliability and familiarity with the accredited pharmacist were the main perceived benefits of this model. However, patient familiarity with the community pharmacist was considered by some GPs to be of greater importance, as unfamiliarity with the HMR pharmacist may result in refusal to participate in the service. Consequently, most GPs indicated that the referral pathway decision should be made by the patient. This preference needs to be considered by pharmacists as many community pharmacists outsource HMRs to accredited pharmacists who work on a contract basis.

This study confirmed that HMRs were perceived as useful educational tools to GPs. GPs also believed that patients feel reassured and feel more independent as a result of being educated by the pharmacist during a HMR. These findings were consistent with the national and international literature on medication reviews [6,10,11] and similar services [17]. The results from this study support previous studies which indicated that HMRs provide a mechanism to identify MRPs and recommend alternative treatment options to resolve problems [9,18]. As such, it provides additional evidence of the perceived value of HMRs in improving clinical outcomes by facilitating safe and effective use of medicines and increased GP and patient knowledge of medicines [12].

Some of the strategies suggested by the GPs to improve the HMR process were consistent with previous studies [6,10,11]. Participants described the HMR process as being lengthy, with tedious paperwork not conducive to GPs’ time constraints. The preferred strategy suggested to streamline the process was the introduction of an electronic communication system. This strategy supports previous findings which indicated that an electronic system would simplify the GP referral process and improve the transfer of documents between GPs and pharmacists [6,10]. GPs supported the concept of a standard HMR reporting template to improve the consistency of reports produced by pharmacists. The frequent reporting of these strategies highlights the lack of awareness on the availability of these resources including the online HMR referral and management form which exist within a commonly used GP’s prescribing software [19]. Measures should be taken to increase awareness and the use of existing HMR tools. Other strategies included assigning personnel, such as a practice nurse manager, to be the focal point of the program to improve efficiency of the process. This finding is similar to other studies [6,10,11]. GPs were of the opinion that intrusion into the patient’s domestic setting could be a barrier. This raises GP concerns of underlying factors such as cultural barriers and domestic issues that may be contributing to a patient’s resistance towards the service. However, a previous study that involved a patient survey indicated that the discomfort patients feel about being visited in the home by a pharmacist was not significant whereas their perception of personal benefit from the HMR was influential in their decision to have a HMR [20].

As the recruitment was limited to GPs who had experience with HMRs, those who volunteered or were recommended may have been biased towards the service. Nonetheless, previous HMR experience was a requirement to ensure that participants had a working knowledge of the service and could provide informed responses. The low uptake of HMRs in WA made recruitment challenging, resulting in the utilisation of snowball sampling.

## Conclusion

While the findings have confirmed that HMRs improve GPs’ knowledge of the medicines their patients are taking and medication management advantages for patients, it also raised issues pertaining to GP convenience and patient familiarity with the accredited pharmacist conducting the review. Resolving both concerns could improve the uptake of the HMR service. There is a need for further research into strategies to overcome these barriers and establish whether the strategies proposed are beneficial towards improving the HMR process.
